# Secondary Prevention of Myocardial Infarction With Nonpharmacologic Strategies in a Medicaid Cohort

**Published:** 2009-03-15

**Authors:** Erica B. Oberg, Annette L. Fitzpatrick, William E. Lafferty, James P. LoGerfo

**Affiliations:** Department of Health Services; University of Washington School of Public Health and Community Medicine, Seattle, Washington; University of Washington School of Public Health and Community Medicine, Seattle, Washington; University of Washington School of Public Health and Community Medicine, Seattle, Washington

## Abstract

**Background:**

The quality of health care after myocardial infarction (MI) may be lacking; in particular, guidelines for nonpharmacologic interventions (cardiac rehabilitation, smoking cessation) may receive insufficient priority. We identified gaps between secondary prevention guidelines and ambulatory care received by Medicaid enrollees after an MI.

**Methods:**

MI survivors were selected by using 2004 Washington State Medicaid administrative claims. Deidentified data were abstracted for hospitalizations, ambulatory care, and prescriptions for 365 days after the MI. Cox regression analysis compared utilization of guideline-directed secondary prevention strategies with death and recurrent hospitalization.

**Results:**

The sample size was 372. Fifty patients died during the year after the MI, and 144 were rehospitalized. Only 2 patients attended a cardiac rehabilitation program. Tobacco cessation counseling was associated with a 66% reduction in death, but only 72.6% of smokers were counseled. Less than half (45.4%) of patients saw a primary care provider within 90 days of their MI, and 7.5% never contacted a health care provider. Receiving regular primary care was associated with a decreased risk for death (hazard ratio, 0.91; 95% confidence interval, 0.84-0.97, *P* < .01). A protective trend was associated with care by a cardiologist, but only 21.5% received specialist care.

**Conclusion:**

Analysis of Medicaid claims data suggests rates of secondary prevention are less than optimal. To improve survival and reduce rehospitalization after an MI, policy changes (tobacco cessation benefits, expansion of rehabilitation programs), health care capacity (training, referral patterns, and coordination of care), and improvements to access (removing barriers, increasing facilities, targeting minority populations) could be implemented.

## Introduction

Cardiovascular disease is the leading cause of death for men and women in the United States. In 2004, approximately 7.9 million Americans had a myocardial infarction (MI); of these, 452,327 or 5.7% died acutely (1). Recurrent MIs are largely preventable by aggressive risk factor reduction, including pharmacologic and lifestyle recommendations. One-year survival improved from 74.7% with no care to 95.7% with optimal care, including pharmacologic and lifestyle recommendations (2). Many organizations, such as the American Heart Association (AHA), American College of Cardiology (ACC), and European Society of Cardiology, have published guidelines that specify the evidence-based components of optimal secondary prevention, and programs such as AHA's Get With the Guidelines help implement those guidelines (3-6). However, evidence suggests discharge planning and outpatient secondary prevention are not improving as rapidly as processes of optimal inpatient care, despite guidelines (7).

In particular, guidelines associated with nonpharmacologic interventions (cardiac rehabilitation, smoking cessation, physical activity, weight reduction) may receive insufficient priority. Recent results from facilities implementing Get With the Guidelines quality improvement programs show nonpharmacologic interventions are recommended 26% to 54% of the time, compared with drug recommendations that were made 86% to 93% of the time (8). Many studies describe aspects of adhering to pharmacologic regimens for secondary prevention, but few have documented rates of lifestyle management after MI (9-12). To our knowledge, none has done so in a Medicaid population. The need for nonpharmacologic strategies is well documented (13). In addition to secondary prevention of MI, lifestyle modifications reduce the risk for many chronic diseases and have been prioritized as a common agenda by the AHA, American Diabetes Association, and American Cancer Society (14).

In addition, many studies demonstrate lower quality of care and poorer MI outcomes in patients of lower socioeconomic status (SES) or minority ethnicities (15-17). Disparities between the care of whites and minorities has been documented in all aspects of care, from the rate at which interventional procedures are offered to ambulatory screening for cardiovascular risk factors (18-20). In fact, the rate of MI-related deaths showed the same small differences at 1 year: 39.7% for blacks and 37.6% for whites (*P* = .001) (21). Minorities are disproportionately represented in lower SES strata; compared with lower-SES patients, more affluent and better-educated patients were more likely to receive cardiac rehabilitation (43.9% vs 25.6%, *P* < .001) or to be seen by a cardiologist (56.7% vs 47.8%, *P* < .001) (22). Some of these disparities may be attributable to underlying differences in access to care (privately insured vs Medicare/Medicaid); however, the ratio of minorities enrolled in Medicaid is substantially higher than in the general population.

For these reasons, and because Medicare/Medicaid programs are publicly funded, we observed ambulatory health care utilization, with emphasis on nonpharmacologic interventions, among Washington Medicaid enrollees during the year after they experienced an MI to better understand the characteristics and quality of care they received. These findings may be useful to address gaps in access to and utilization of secondary prevention programs that include nonpharmacologic strategies.

## Methods

This study included all Medicaid enrollees diagnosed with an MI and discharged alive from an inpatient facility in 2004, on the basis of administrative claims data from the Washington State Department of Social and Health Services. Institutional review board approval was obtained from both the state of Washington and the University of Washington. Data consisted of deidentified recipient and claims information (medical and pharmacy) for Medicaid fee-for-service enrollees discharged from an inpatient facility with a diagnosis of MI (International Classification of Diseases, Ninth Revision codes 410.xx) during 2004. Excluded from the sample were any patients who were not continuously eligible for Medicaid insurance coverage for 365 subsequent days, unless the person died while eligible. Of an eligible sample of 395 patients, we excluded 15 who did not have claims beyond the first 7 days after discharge and 8 who were missing data such as Medicare eligibility status (Figure). Most analyses are based on this sample of 372; for analyses involving details of subsequent diagnoses (such as comorbidity), the completeness of the data limited the sample to single-eligibility Medicaid enrollees. The details of claims were abstracted for the initial MI hospitalization, any rehospitalizations, any ambulatory care, and any prescriptions that the patient received during the 365 days after the MI. To create an analyzable dataset, 3 separate data files that contained multiple lines per case were aggregated into single variables by using AHA/ACC guidelines for secondary prevention.

**Figure.**
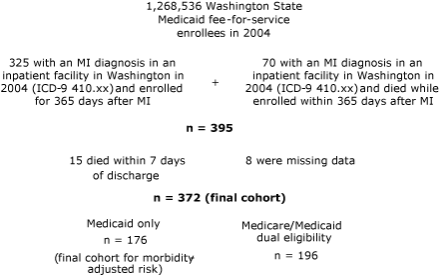
Sample selection of 372 Washington State Medicaid or Medicare/Medicaid recipients who had a diagnosis of MI in 2004. Abbreviations: MI, myocardial infarction; ICD-9, International Classification of Diseases, Ninth Revision.

Nonpharmacologic risk factor reduction strategies are difficult to capture with claims data because of incomplete documentation. Consequently, we included only variables that were reimbursed by Medicaid and therefore most likely to be accurately documented; this limited variables to cardiac rehabilitation and smoking cessation (23). Cardiac rehabilitation services were identified by Diagnosis-Related Group or Current Procedural Terminology (CPT) codes. The CPT definitions are used by the Centers for Medicare and Medicaid Services to reimburse for cardiac rehabilitation and usually represent the method that other payers, including the Department of Social and Health Services, would likely use. Smoking cessation was defined by using both International Classification of Diseases diagnoses and supplemental classification codes and CPT codes for basic or intensive counseling. We did not identify prescription smoking cessation aids because Medicaid reimbursement limits this to buproprion, which may be prescribed for other indications.

Ascertaining comorbidity was difficult because of variable levels of detail among claims; hospitalizations could include up to 9 concomitant diagnoses, ambulatory visits had up to 4 diagnoses, and claims for dually eligible enrollees variably contained between 0 and 4 diagnoses. Because of this variation, a count of comorbid conditions was unreliable. We excluded symptom-based and self-limiting diagnoses, and after ranking by frequency, we selected diagnoses associated with chronic diseases: heart failure, cancer, renal failure, or diabetes. These were included as a covariate in models in an attempt to adjust for severity of illness. Because of the imperfect nature of this definition and because of incomplete ascertainment of comorbidity in the dually eligible Medicare/Medicaid enrollees, analyses adjusted for comorbidity were run only on the Medicaid-only split sample.

Data were analyzed by using SPSS version 11 (SPSS, Inc, Chicago, Illinois). To characterize care received by Washington Medicaid enrollees who were hospitalized for MI in 2004, descriptive statistics were generated for demographic characteristics, Medicaid/Medicare eligibility status, and comorbidity. We compared Medicaid-only with Medicare/Medicaid enrollees by using *t* tests and χ^2^ analyses. To investigate associations between guideline-directed secondary prevention strategies and recurrent hospitalization or death within the 12 months after MI, we developed Cox proportional hazard models that used time to death or time to first rehospitalization as dependent variables. The analyses were run both unadjusted and adjusted for significant variables: age, sex, ethnicity, and comorbidity.

## Results

Enrollees were predominantly white urban residents; a slightly larger percentage were men, and the average age was 64 years (Table 1). Women were significantly older than men when they had their MI (67 years for women, 60 years for men, *P* < .001). The patients represented 2 broad categories in terms of Medicaid eligibility. Slightly less than half (47.3%) had Medicaid coverage only; the remainder (52.7%) had coverage under both Medicare and Medicaid. As would be expected, patients with both Medicare and Medicaid coverage were significantly older than those who had only Medicaid coverage. Single-eligibility patients were significantly more likely to be nonwhite. The 2 groups did not differ significantly in terms of residence.

For each patient, we obtained details from claim records regarding the quantity and type of medical care they obtained in the year after their MI. In total, 38.7% of patients were rehospitalized within a year; single-eligibility patients had significantly fewer rehospitalizations than did Medicaid/Medicare patients, although this finding was expected because dually eligible patients were older. Of the 372 patients, 50 died during the year after their MI (Table 1).

More than 80% of the patients had diagnoses related to the circulatory system, including 30% with subsequent MI, 14% with other acute and subacute forms of ischemic heart disease, 25% with previous MI, and 48% with other forms of chronic ischemic heart disease (up to 9 diagnoses were documented per hospitalization). Half of the patients had diagnoses involving endocrine, nutritional, and metabolic diseases, including diabetes (29%), disorders of lipid metabolism (25%), and obesity (10%). Sixteen percent had tobacco-use disorder diagnoses. Only 2 patients participated in a cardiac rehabilitation program, even though rehabilitation is covered by Medicaid. Smoking cessation counseling was more widely offered; 73% of smokers received smoking cessation counseling at least once. Counseling was associated with a 66% mortality reduction (*P* = .08) in the crude Cox regression model. The effect of cardiac rehabilitation on outcomes of interest could not be tested because of the small number of patients who received it, although data from large clinical trials have demonstrated substantial benefit (24,25).

Having a primary care visit during the first 90 days after an MI was associated with a lower risk of rehospitalization (Table 2); however, the opposite effect was seen with risk of death (Table 3). When adjusted for demographic characteristics and comorbidity, the association with risk of death was attenuated and no longer significant. A total of 28 patients (7.5%) had no follow-up care at all during the year after their MI, although each had at least 1 claim for medications or laboratory services.

## Discussion

### Reducing risk factors

Adherence to cardiovascular disease prevention guidelines improves survival, reduces recurrent events and the need for interventional procedures, and improves the quality of life. The magnitude of risk reduction seen in the literature ranges from a 12% reduction in mortality associated with aspirin use, to a 25% mortality reduction over 2 years with participation in a cardiac rehabilitation program, to an estimate of nearly 50% fewer deaths from coronary artery disease over 2 years if guidelines were followed in all cases (26). Attempts to quantify the benefit of rehabilitation beyond adherence to drug regimens estimated a 52% reduction in reinfarction (27).

Rates of cardiac rehabilitation in the United States are generally low despite these benefits. Estimates of national utilization rates have ranged from 9% to 34% (28,29). Data specific to the state of Washington, where the study sample resided, are limited to the patient self-reported measures collected in the Behavioral Risk Factor Surveillance System Survey; 28% of Washingtonians who had a heart attack or stroke reported participating in a rehabilitation program (30). Our findings suggest a lower rate of rehabilitation among Medicaid enrollees. This rate may be low for several reasons. As noted previously, rates of provider recommendations for nonpharmacologic interventions are low; few Medicaid enrollees may have been referred to cardiac rehabilitation. Additionally, minorities are overrepresented in the Medicaid population; our findings may reflect the lower quality of care and poorer MI outcomes typically seen among patients of lower socioeconomic status or minority ethnicities (15-17).

Tobacco cessation is a priority according to secondary prevention guidelines, although rates of tobacco use in our sample were below the national average. Nationally, 29% of Medicaid enrollees smoke (31). Among Washington Medicaid enrollees, the rate of documented tobacco use was 19%. Of course, this statistic is subject to limitations in coding; that is, providers are more likely to document use if they are planning to counsel the patient about cessation. Claims data cannot capture instances in which smoking status was not obtained. However, we found a significant association between tobacco cessation advice and survival. During hospitalization for MI, smokers are generally offered nicotine patches. However, after discharge, this prescription is not routinely continued because nicotine patches are not covered by many insurance plans, including Medicaid. Simple changes in discharge planning and benefits structure could ensure that MI survivors who quit smoking in the hospital remain tobacco-free after discharge by using aids such as nicotine patches.

### Health care utilization

Although secondary prevention guidelines do not specify an optimal schedule of outpatient management, reestablishing prompt contact with a primary care provider is considered essential to continuity of care. Cardiology specialty care may be appropriate for most patients after an MI, especially if a stent was placed or if the patient underwent coronary bypass surgery or another invasive procedure. Our findings concerning the positive effect of primary care are limited by the information available in the dataset, but they suggest a contribution to outcomes that has also been seen in other studies (32). We saw a reduced adjusted hazard ratio associated with prompt primary care that lost significance when adjusted for comorbidity. This finding is most likely because primary care providers usually see patients with more comorbidities (33). Some of the reduced risk may be associated with primary care providers' correction of medication regimens that were incomplete at discharge (34).

The role of specialty care is clearer; cardiologists make more referrals to cardiac rehabilitation programs, follow guidelines more closely, and their patients have better survival rates overall (32,33,35), although the survival benefit associated with cardiology care (as opposed to care delivered by primary care providers) disappears when adjusted for comorbidity and optimal adherence to medications (35). In our study, 21.5% of patients saw a cardiologist during the year of follow-up; among these patients, cardiology care showed a nonsignificant trend toward a survival benefit. However, our ascertainment of the severity of the initial MI was limited to diagnoses codes, and severity of the MI would be relevant in determining if all patients were in need of specialized care by a cardiologist or if their care could be appropriately managed by a primary care provider. Cardiology care was associated with an increased rehospitalization rate, which may be due to more admissions for nonurgent invasive procedures or increased severity of the MI.

Insurance status affects the quality of hospital care; Medicaid patients receive fewer cardiac procedures and have higher mortality (36-38), which may be true of the Medicaid population we studied as well. These results should be of special interest for those concerned about reducing disparities in medical care for low-income, ethnically diverse populations.

Our analysis of nonpharmacologic strategies was limited to cardiac rehabilitation and smoking cessation because of limitations in the data in Medicaid administrative claims. Many other aspects of secondary prevention are important as well. We assess the prescription of and adherence to medications in the same population elsewhere (39). However, future studies could use methods other than claims to better detect rates of physical activity recommendation or weight management.

Secondary prevention strategies are effective, and trends in our data confirm that observation. Utilization of nonpharmacologic strategies among Medicaid enrollees is less than optimal, possibly for reasons related to access and delivery of health care. To improve survival and reduce recurrent hospitalization after an MI, the access and delivery of health care could be changed in ways that expand secondary prevention. These include offering more tobacco cessation assistance, implementing more cardiac rehabilitation programs, and automatically referring patients to such programs. Providers could be trained to refer patients to these programs more frequently, or in areas where formal rehabilitation programs are not easily accessed, lifestyle change counseling may improve outcomes. Particular emphasis should be placed on increasing referrals for women and minorities, who are less frequently referred to rehabilitation programs (28,29). Improving the coordination of care between primary care providers and inpatient facilities may reduce the number of patients who never follow up with primary care. Barriers to participation in secondary prevention include many socioeconomic factors (28). Improvements to health care delivery should be undertaken in concert with community-based efforts to reduce barriers to utilization and increase awareness among patients about the benefits of secondary prevention in preventing future events and rehospitalizations.

## Figures and Tables

**Table 1:** Demographic Data for Washington State Medicaid Recipients Who Survived a Myocardial Infarction in 2004

**Characteristic**	All Patients (N = 372)	Medicaid Only (n = 176, 47.3%)	Medicare/Medicaid Dual Eligibility (n = 196, 52.7%)	*P* Value^a^
**Mean age (SD)**	64 (13.5)	56 (10.9)	70 (11.9)	<.001
**Sex, n (%)**				
Male	191 (51.3)	101 (57.4)	90 (45.9)	.03
Female	181 (48.7)	75 (42.6)	106 (54.1)	
**Race/ethnicity, n (%)**				
White	250 (67.2)	104 (59.1)	146 (74.5)	.002^b^
Nonwhite	122 (32.8)	72 (40.9)	50 (25.5)	
African American	27 (7.3)	24 (13.6)	3 (1.5)	
Asian American	29 (7.8)	14 (8.0)	15 (7.7)	
American Indian	11 (3.0)	7 (4.0)	4 (2.0)	
Hispanic	22 (5.9)	11 (6.3)	11 (5.6)	
Other/missing	33 (8.8)	16 (9.1)	17 (8.7)	
**Residence, n (%)^c^**				
Metropolitan/urban	294 (79.1)	144 (81.8)	150 (76.5)	.24^c^
Micropolitan	37 (10.0)	15 (8.5)	22 (11.2)	
Small town	21 (5.7)	8 (4.5)	13 (6.6)	
Rural	19 (5.1)	8 (4.5)	11 (5.6)	
**Deceased, n (%)**	50 (13.4)	14 (8.0)	36 (18.4)	.003
**Recurrent hospitalization, n (%)**	144 (38.7)	93 (52.8)	51 (26.0)	.001
**Mean no. of rehospitalizations during the year (SD)**	1.1 (2.3)	1.5 (2.5)	0.4 (1.2)	<.001
**Mean no. of days to first rehospitalization (SD)**	118.6 (102.3)	95 (96.9)	161.6 (98.6)	<.001
**Received an invasive procedure within the year, n (%)**	95 (25.5)	89 (50.6)	6 (3.1)	<.001
**Comorbidity at time of MI, n (%)**	113 (30.4)	104 (59.1)	9 (4.6)	<.001
**Documented tobacco use, n (%)**	71 (19.1)	68 (38.6)	3 (1.5)	<.001

Abbreviations: SD, standard deviation; MI, myocardial infarction.

a Calculated by using Fisher exact *t* test (when cell size was small) and χ^2^ tests.

b *P* value is for difference between whites and all other racial/ethnic minorities combined because of small numbers in each race/ethnicity category.

c *P* value is for difference between urban and nonurban residence only. Rural-Urban Commuting Area codes classify US census tracts by using measures of population density, urbanization, and daily commuting. One Medicaid recipient in the sample was missing data on residence.

**Table 2:** Nonpharmacologic Health Care Utilization and Rehospitalization Among 372 Medicaid Recipients Who Survived a Myocardial Infarction, Washington State, 2004

**Aspect of Care^a^**	Crude HR for Rehospitalization in 1 Year (95% CI)	*P* Value	Model 1, Adjusted HR for Rehospitalization in 1 Year (95% CI)^b^	*P* Value	Model 2, Adjusted HR for Rehospitalization in 1 Year (95% CI)^b^	*P* Value
Saw PCP within 90 days (n = 169)	0.69 (0.54-0.90)	<.01	0.72 (0.55-0.93)	.01	0.75 (0.50-1.13)	.17
No. of PCP visits (mean, 11.3)	1.02 (1.01-1.03)	<.01	1.01 (1.00-1.03)	<.01	1.01 (1.00-1.03)	.06
Saw cardiologist within 1 year (n = 80)	1.38 (0.97-1.98)	.08	1.03 (0.81-1.06)	.25	1.14 (0.74-1.76)	.54
No. of cardiology visits (mean, 1.3)	1.04 (1.01-1.06)	<.01	1.03 (1.01-1.06)	<.01	1.02 (1.0-1.05)	.10
Received smoking cessation counseling at least once (n = 53)	1.55 (1.08-2.22)	.02	1.39 (0.94-2.07)	.10	1.20 (0.77-1.87)	.41

Abbreviations: HR, hazard ratio; CI, confidence interval; PCP, primary care provider.

a Only 2 Medicaid recipients attended a cardiac rehabilitation program during the year after myocardial infarction. This number was too small to calculate HRs for rehospitalization.

b Model 1 adjusted for age, sex, and race; model 2 also adjusted for comorbidity for Medicaid-only patients (n = 176).

**Table 3:** Nonpharmacologic Health Care Utilization and Survival Among 372 Medicaid Recipients Who Survived a Myocardial Infarction, Washington State, 2004

**Aspect of Care^a^**	Crude HR for Death in 1 Year (95% CI)	*P* Value	Model 1, Adjusted HR for Death in 1 Year (95% CI)^b^	*P* Value	Model 2, Adjusted HR for Death in 1 Year (95% CI)^b^	*P* Value
Saw PCP within 90 days (n = 169)	2.07 (1.40-3.08)	<.01	1.64 (1.11-2.42)	.01	1.48 (0.49-3.89)	.54
No. of PCP visits (mean, 11.3)	0.90 (0.85-0.95)	<.01	0.92 (0.87-0.97)	.51	0.91 (0.84-0.97)	.005
Saw cardiologist within 1 year (n = 80)	0.47 (0.20-1.10)	.08	0.75 (0.31-1.81)	.52	0.68 (0.18-2.53)	.57
No. of cardiology visits (mean, 1.3)	0.99 (0.92-1.06)	.72	1.01 (0.95-1.08)	.76	1.01 (0.95-1.08)	.67
Received smoking cessation counseling at least once (n = 53)	0.44 (0.17-1.11)	.08	0.89 (0.33-1.81)	.81	0.99 (0.29-4.43)	.98

Abbreviations: HR, hazard ratio; CI, confidence interval; PCP, primary care provider.

a Only 2 Medicaid recipients attended a cardiac rehabilitation program during the year after myocardial infarction. This number was too small to calculate HRs for death.

b Model 1 adjusted for age, sex, and race; model 2 also adjusted for comorbidity for Medicaid-only patients (n = 176).
